# Determination and Disposition of the Aromatase Inhibitor Exemestane in CYP3A-Deficient Mice

**DOI:** 10.3390/molecules30071440

**Published:** 2025-03-24

**Authors:** Hanieh Taheri, Eman Ahmed, Peng Hu, Alex Sparreboom, Shuiying Hu

**Affiliations:** Division of Pharmaceutics and Pharmacology, College of Pharmacy, The Ohio State University, Columbus, OH 43210, USA; taheri.13@osu.edu (H.T.); ahmed.1080@osu.edu (E.A.); hu.1130@osu.edu (P.H.); sparreboom.1@osu.edu (A.S.)

**Keywords:** aromatase inhibitors, exemestane, mouse plasma, pharmacokinetics, LC-MS/MS

## Abstract

Exemestane, a steroidal aromatase inhibitor prescribed for post-menopausal women with estrogen receptor-positive breast cancer, is associated with debilitating musculoskeletal side effects and exhibits considerable interindividual variability in systemic exposure. Although exemestane is metabolized mainly by cytochrome P-450 3A4, the contribution of this metabolic pathway to the elimination of exemestane and its drug–drug interaction liabilities remains uncertain. Here, we developed a novel quantification method for exemestane and applied it to evaluate the role of CYP3A-mediated metabolism in the pharmacokinetics of exemestane using wild-type and *Cyp3a*-deficient mice. Liquid chromatography–mass spectrometry was used to quantify exemestane in selective reaction monitoring (SRM) mode, in which precursor ion and fragment ion data were obtained simultaneously. Validation results demonstrated that the developed method was accurate and precise, and sufficiently sensitive to be applied to murine pharmacokinetic studies involving serial blood sampling strategies. Although in vitro studies indicate that exemestane undergoes extensive metabolism in the liver to inactive metabolites by CYP3A4, complete *Cyp3a* deficiency in mice did not influence the systemic exposure to exemestane. This unequivocal evidence from genetic approaches using preclinical mouse models confirms that the potential for such DDI liabilities is very low. Our newly developed method provides a robust platform for further pharmacokinetic studies with exemestane in mice to delineate DDI liabilities and define the mechanisms of elimination.

## 1. Introduction

Exemestane (Aromasin) is a steroidal aromatase inhibitor commonly prescribed to post-menopausal women with estrogen receptor-positive breast cancer at a dosage of 25 mg/day [1,2,3,4]. Aromatase, the enzyme targeted by exemestane, converts androgens to estrogens, and plays a crucial role in estrogen synthesis in post-menopausal women [5,6,7,8]. Overexpression of aromatase in stromal and parenchymal carcinoma cells enhances estrogen access, leading to excessive proliferation [6,9]. Exemestane irreversibly binds to the substrate binding site on the aromatase enzyme, resulting in its permanent degradation [3,5]. Aromatase inhibitors, including exemestane, can suppress over 98% of aromatase activity, leading to a more than 90% reduction in estrogen plasma levels [6,10]. Unfortunately, the clinical use of exemestane is associated with debilitating side effects, especially musculoskeletal symptoms such as arthralgia, myalgia, and joint/muscle stiffness, which are observed in 20–74% of patients [3,6,11,12]. These side effects can adversely impact the quality of life of patients and potentially lead to non-adherence or discontinuation of treatment [6,11,13].

Similar to the heterogeneity observed in the manifestation of side effects, exemestane displays significant interindividual pharmacokinetic variability [4,14,15,16,17], presumably due to variation in the function of enzymes and transporters involved in the absorption and disposition of exemestane. After oral administration, exemestane undergoes extensive CYP3A4-mediated metabolism to form the inactive oxidized metabolite exemestane 6-hydroxymethylexemestane (MII), with a minor contribution from alternative metabolic pathways involving aldoketo reductase (AKR) and CYP4A11/CYP1A to form the active metabolite 17-hydroexemestane (MI), which is further metabolized by CYP3A and UGT2B17 to inactive oxidized and glucuronidated metabolites [16,18,19,20,21,22]. Levels of the unchanged drug in plasma only account for less than 10% of the total drug, and the metabolites are either inactive or inhibit aromatase with decreased potency compared with the parent drug. Despite the positive association reported in patients carrying a reduced function single-nucleotide polymorphism (SNP) in CYP3A4 with higher exemestane concentrations [23], coadministration of ketoconazole, a potent inhibitor of CYP3A4, has no significant effect on exemestane pharmacokinetics [19,24]. Given that no other formal DDI studies have been conducted, there are uncertainties regarding the in vivo importance of this pathway, with paradoxical observations regarding the influence of potent inhibitors and inducers having been reported. To resolve these apparent discrepancies, we explored genetic approaches using preclinical mouse models and provide evidence that the potential for such DDI liabilities is very low. Since existing quantification methods for exemestane have primarily focused on the use of large sample volumes and human studies, such methods may not provide the sensitivity and reliability required for mouse studies where sample volumes are limited. To address this issue, we report here the development and validation of an analytical method for the quantification of exemestane in mouse plasma and its implementation in pharmacokinetic studies in wild-type mice and mice lacking all *Cyp3a* isoforms [*Cyp3a(-/-)*].

## 2. Results and Discussion

### 2.1. Chromatographic and Mass Spectrometric Conditions

**Figure 1:**
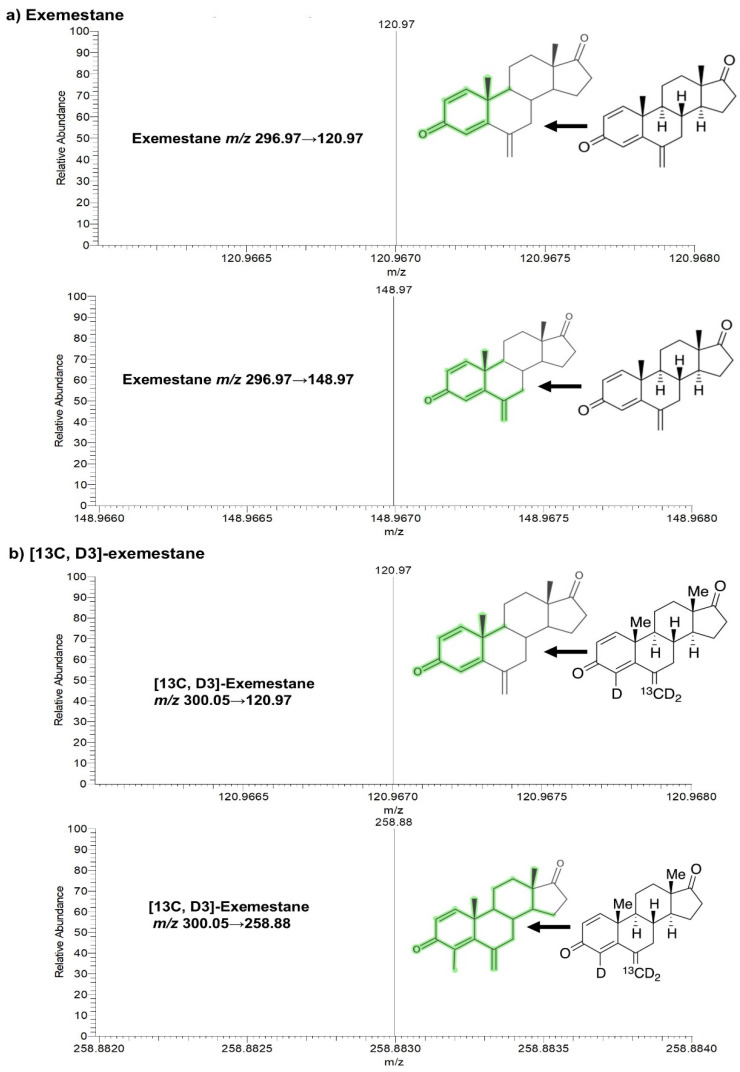
Fragmentation and SRM scanning spectra of (**a**) exemestane and (**b**) [13C, D3]-exemestane. The portion of the molecule highlighted in green indicates the fragment ion.

**Figure 2:**
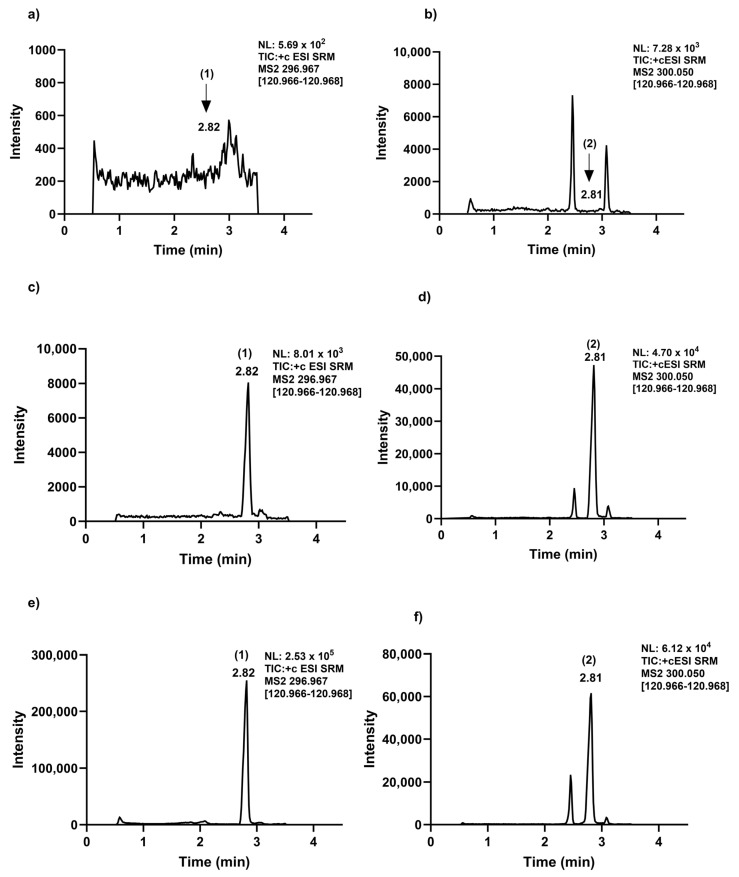
Chromatograms of exemestane (1) and [13C, D3]-exemestane (2) in mouse plasma: monitoring (**a**) exemestane in the untreated mouse plasma (the arrow at 2.82 min) and (**b**) [13C, D3]-exemestane in the untreated mouse plasma (the arrow at 2.81 min); (**c**) 0.4 ng/mL (LLOQ) exemestane spiked into the untreated mouse plasma; (**d**) 2 µg/mL [13C, D3]-exemestane spiked into the untreated mouse plasma; (**e**) exemestane detected in treated sample collected at the time point of 15 min; and (**f**) 2 µg/mL [13C, D3]-exemestane spiked into the treated sample processed according to the sample preparation. Peak (1) is exemestane and peak (2) is [13C, D3]-exemestane. The arrow shows the elution point of exemestane and [13C, D3]-exemestane in the untreated matrix.

An ESI source running in the positive mode was used to ionize exemestane and its internal standard. Exemestane was detected in the SRM mode at *m*/*z* 297.0 → 121.0 (as quantifier) and 297.0 → 149.0 (as qualifier), consistent with a previously reported protocol [15,25,26], and 300.1 → 121.0 (as quantifier) and 300.1 → 258.9 (as qualifier) for [13C, D3]-exemestane (Table 1 and Figure 1). The mass spectrometer parameters were optimized and configured as follows: 25 arbitrary units (Arb) for sheath gas, 5 Arb for auxiliary gas, and 1 Arb for sweep gas. The temperature of the ion transfer tube and vaporizer were adjusted to 340 °C and 360 °C, respectively. The system was operated using heated electrospray ionization (ESI) as the ion source. The ion spray voltage was adjusted to 3600 V for the analysis of exemestane. Argon was used as the collision gas and was maintained at a pressure of 1.5 mTorr (Table 1). In order to optimize symmetry of peaks, resolution, retention time, and separation from potentially interfering endogenous substances, various compositions of mobile phase B and modifiers, as well as the temperature of the column, were examined in multiple iterations. Exemestane and [^13^C, D_3_]-exemestane signal responses were evaluated concurrently utilizing two additives, 0.1% formic acid and 0.1% acetic acid, introduced into the aqueous phase reagent. For subsequent steps, 0.1% acetic acid was preferred since it provided a noticeably increased signal response for exemestane. Under optimal conditions, the average retention time for exemestane was 2.82 min and 2.81 min for [^13^C, D_3_]-exemestane (Figure 2c,d). It was possible to distinguish and separate the endogenous peaks from the internal standard at a flow rate of 0.3 mL/min (Figure 2b,d,f).

In order to separate analytes from endogenous interferences and improve sensitivity, two reversed-phase HPLC columns were examined: an Acquity UPLC BEH C18 (50 mm × 2.1 mm, 1.7 µm) and an Accucore aQ (50 mm × 2.1 mm, 2.6 μm). Because of the successful baseline separation of analytes, the shorter run time, and the achievement of symmetrical chromatographic peaks, the Acquity UPLC BEH C18 column was selected for further analyses. Moreover, Acquity UPLC BEH C18 provided a superior sensitivity with an LLOQ of 0.4 ng/mL, which is 25 times lower than that accomplished with the Accucore aQ column (LLOQ of 10 ng/mL).

Regarding the selection of an appropriate stationary phase, a previous study reported the presence of interfering peaks near the elution of exemestane. To improve selectivity, a core-shell column was used to help achieving an LLOQ of 0.5 ng/mL with 100 µL of human plasma [27]. Previous studies have also demonstrated that further increases in sensitivity (LLOQ of 0.05 ng/mL) can be achieved by employing 0.5 mL of plasma [28], although such volumes are not suitable for murine pharmacokinetic studies involving repeat sampling approaches. To date, six LC-MS/MS analytical methods for exemestane measurement have been reported in which the LLOQ has ranged from 0.05 to 7.0 ng/mL [15,26,27,28,29,30]. These studies have all centered on analyzing the agent in human plasma or urine [29]. Although no methods exist for application to mouse plasma, exemestane was previously quantified in rat sample using a method with an LLOQ of 1 ng/mL that requires 100 µL of rat plasma [31]. To generate a universally applicable procedure with sufficient sensitivity that is adaptable to small sample volumes associated with large-scale preclinical pharmacokinetic investigations [15,31], we set out to develop an analytical method with an LLOQ of 0.4 ng/mL requiring only 10 µL of mouse plasma.

### 2.2. Method Validation

#### 2.2.1. Exemestane Selectivity and Linearity

The Xcalibur software (version 4.4.16.4) automatically plotted exemestane calibration curves spanning the range of 0.4–75 ng/mL using 1/x^2^ weighted least-squares linear regression. Over the course of four days, two calibration curves were constructed every day, and these showed satisfactory linearity with a coefficient of determination (r2) of >0.997. The LLOQ for exemestane was determined to be 0.4 ng/mL. The typical SRM chromatograms of exemestane along with the appropriate internal standard from untreated mouse plasma and LLOQ are shown in (Figure 2a,b) and (Figure 2c,d), respectively. When comparing the exemestane retention time in blank mouse plasma to the corresponding chromatograms of LLOQ samples, no endogenous interference was seen (Figure 2a,c), and successful resolution and separation was achieved between potentially interfering endogenous compounds with [13C, D3]-exemestane (Figure 2b,d). In the treated mouse plasma sample, exemestane was detected (Figure 2e) and the [13C, D3]-exemestane was spiked and processed according to the sample preparation (Figure 2f).

#### 2.2.2. Exemestane Precision and Accuracy

The observed intra- and inter-day precision results associated with LLOQ samples for exemestane were 7.09% and 4.64%, respectively, with an accuracy (bias, %) of 5.10% (Table 2). Four QC levels (LQC, MQC, HQC, and AULQ) were found to have an intra-day precision of ≤3.67% and an inter-day precision of ≤4.93%, with an overall accuracy ranging from −7.80% to −2.50% (Table 2), thereby satisfying the pre-defined guidelines for the validation of bioanalytical methods (i.e., <15%). The precision and accuracy results for exemestane in 10-fold diluted AULQ suggest that, following dilution with blank plasma to ensure levels fall within the calibration range, an unknown sample with a concentration exceeding the selected ULQ can also be accurately and reliably quantified (Table 2).

#### 2.2.3. Matrix Effect, Extraction Recovery, and Carryover

Matrix effects were evaluated at three designated QC samples, exhibiting values ranging from 96.9% to 108% (≤3.74%, CV%) (Table 3). The influence of hemolysis on assay performance was evaluated at the same QC samples, exhibiting 92.9% to 102% (≤10.3%, CV%) (Table 3). These findings imply that the method can accurately quantify exemestane in mouse plasma over the chosen quantitative range irrespective of sample hemolysis and with the knowledge that matrix effects are negligible. Moreover, the analyte could be effectively and reproducibly extracted from mouse plasma using a one-step protein precipitation method. The percentage deviation of all LLOQ samples, following injection of an ULQ sample, was always less than 20%, suggesting that any potential carryover related to the instrument and/or the column is minimal. This finding is consistent with the notion that residual exemestane was not detectable in zero calibrators. The recovery values of exemestane extraction were computed at three different QC levels (low, medium, and high) and were found to range from 99.9% to 88.4%, with a variance of ≤8.80% (Table 3).

#### 2.2.4. Stability

##### Short-Term Stability

The stability of exemestane in human plasma and whole blood was previously reported [15,26]. We further explored the stability of the analyte in mouse plasma to simulate several experimental conditions that may occur during sample analysis in preclinical pharmacokinetic studies. In particular, we found that the re-injection stability (12 h at 4 °C), thermostatic autosampler storage (24 h at 4 °C), and bench-top storage (3 h and 6 h at 25 °C ) did not substantially affect the assay performance, with the observed values under those conditions all being within acceptable levels (i.e., >85% compared with corresponding baseline concentrations at time zero) (Table 4 (a)). After 3 h at 37 °C, the drug exhibited signs of degradation, with complete loss of detectability observed after 6 h (Table 4 (b)).

##### Freeze–Thaw Stability and Long-Term Stability

The results of three freeze/thaw cycles (from −80 °C to room temperature/24 h) and long-term stability (5 months at −80 °C) are shown in Table 5 and demonstrate that the storage procedures are adequate (i.e., >85% compared to nominal baseline). These results demonstrate that during sample preparation and routine analysis, exemestane exhibited negligible degradation, thereby ensuring reliable quantification under the applied conditions.

### 2.3. Pharmacokinetic Studies

**Figure 3:**
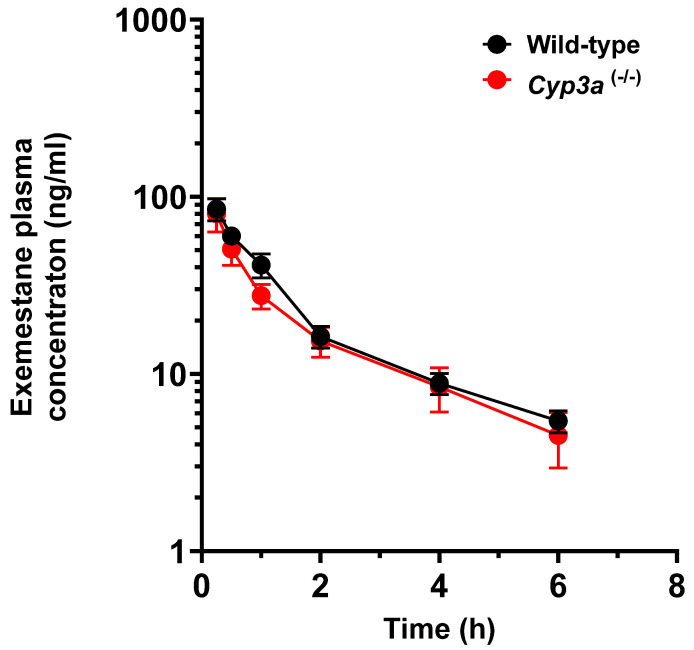
Quantification of the exemestane concentration in mice. Plasma concentrations of exemestane in wild-type and *Cyp3a* (-/-) mice receiving exemestane (20 mg/kg, p.o., N = 10 per genotype). Samples were analyzed using LC-MS/MS.

The newly developed LC/MS-MS method was applied to quantify plasma concentrations of exemestane in mice receiving exemestane orally at a dose of 20 mg/kg (Figure 3). The systemic exposure to exemestane, as estimated from the observed C_max_ and AUC values, were similar in wild-type mice and mice lacking Cyp3a enzymes (Table 6). The results obtained under *Cyp3a*-deficient conditions are consistent with data contained within the prescribing information, suggesting that co-administration with ketoconazole, a potent CYP3A4 inhibitor, does not result in substantial changes in the levels of exemestane in plasma [18]. These collective observations are somewhat unexpected in light of the previous finding from in vitro studies that CYP3A isoforms are the primary enzymes responsible for the formation of the inactive oxidized metabolites [18]. It is conceivable that genetic deficiency or pharmacological inhibition of the pathway results in the shunting of elimination without directly influencing levels of the parent drug [18]. While this provides a plausible explanation for the present observations in *Cyp3a*-deficient mice, this possibility is difficult to reconcile with previously reported pharmacogenetic association studies suggesting that the reduced function variant CYP3A4*22 (rs35599367) is associated with a 54% increase in exemestane concentrations in the plasma of post-menopausal patients with breast cancer [23]. Further studies involving the use of humanized transgenic animals with hepatic expression of functional and nonfunctional human CYP3A variants, analogous to those recently reported by us [32], are currently ongoing to define the contribution of this metabolic pathway to the elimination of exemestane and predict its drug–drug interaction liabilities.

## 3. Materials and Methods

### 3.1. Chemical and Reagents

For method development and animal studies, exemestane (>99.9% purity) and the stable isotope-labeled internal standard [13C, D3]-exemestane (>96.0% purity) were supplied by Toronto Research Chemicals Inc. (North York, ON, Canada) and Selleckchem (Houston, TX, USA), respectively. LC-MS grade acetic acid, formic acid, methanol, acetonitrile (ACN), ammonium acetate, deionized water, dimethylsulfoxide (DMSO), polysorbate 80 (Tween 80), and polyethylene glycol 300 (PEG300) were purchased from Fisher Scientific (Fair Lawn, NJ, USA). Whole blood was obtained from untreated FVB wild-type mice (Taconic Biosciences, Cambridge City, IN, USA), and collected into 1.5 mL heparinized tubes. After centrifugation for 10 min at 4 °C at 6000 RCF, the supernatant layer was collected as blank plasma and stored frozen until needed.

### 3.2. Instrumentation and Mass-Spectrometric Conditions

The analysis was conducted using a Vanquish LC coupled with a Quantiva triple quadrupole mass spectrometer (Thermo Fisher Scientific, FL, USA). Chromatographic separation of analytes was achieved using an Acquity UPLC BEH C18 column (130 Å, 1.7 µm, 2.1 mm × 50 mm, Waters Co.) protected with Acquity UPLC BEH Shield RP18 VanGuard pre-column (130 Å, 1.7 µm, 2.1 mm × 5 mm, Waters Co., MA, USA). The mobile phases A and B and the gradient elution program are provided in Table 1. In order to reduce the possibility of contaminants entering the detector, a diverter valve was used to direct flow into the mass spectrometer for the first 0.5 to 3.5 min of operation and diverted to waste for the remaining time intervals.

The optimal conditions for mass spectrometry were achieved by maximizing the stable response of both the internal standard and the analyte. To achieve this, a standard mixture of exemestane and its internal standard in acetonitrile was infused utilizing a positive ionization mode at a concentration of 1 μg/mL. Table 1 displays the optimized and configured mass spectrometer parameters. The ion source for the system was operated using heated electrospray ionization (ESI). Scheduled selective reaction monitoring (SRM) was used, together with the optimized SRM transitions and associated collision energies shown in Table 1, to analyze exemestane and its internal standard. The data were acquired and processed using Thermo Scientific Xcalibur (version 4.4.16.14; Thermo Fisher Scientific).

### 3.3. Calibration Standards and Quality Control Samples

A 1 mg/mL concentration of exemestane and its internal standard were prepared in DMSO and used as stock solutions. On the day of analysis, independent working solutions were made by diluting the exemestane stock solution with acetonitrile and were then used to prepare the QC samples and calibration standards in mouse plasma. The calibration curve was composed of eight non-zero calibrators over a concentration range of 0.4–75 ng/mL (0.4, 1.5, 3, 5, 10, 25, 50, and 75 ng/mL). Five distinct concentration levels were created as samples for quality control (QC). These levels included the lower limit of quantification (LLOQ), a low-quality control (LQC), a medium-quality control (MQC), a high-quality control (HQC), and a QC above the upper limit of quantification (AULQ). The exemestane concentrations in corresponding QC samples were 0.4, 1.2, 40, 65, and 650 ng/mL. Before sample preparation, the AULQ samples were diluted 10 times with blank mouse plasma to test the dilution integrity. An internal standard working solution was prepared at a concentration of 2 μg/mL by diluting the stock solution with ACN. All solutions were stored at -20 °C and equilibrated to room temperature before use.

The ratio of the analyte peak area to the corresponding internal standard peak area was plotted against the analyte standard concentration to create standard curves. The analyte concentrations were determined by measuring the peak area ratios of the analyte to the internal standard and then calculated from the appropriate standard curve using the Thermo Scientific Xcalibur software (version 4.4.16.14; Thermo Fisher Scientific).

### 3.4. Sample Extraction Procedure

A one-step protein precipitation process was used to extract exemestane from mouse plasma. Frozen samples were thawed and allowed to equilibrate at room temperature before an aliquot of 10 μL was placed into a 0.5 mL Eppendorf tube. To this aliquot were added 5 µL of internal standard working solution and 85 μL of acetonitrile, followed by vortex mixing for 30 sec. Following centrifugation at 16,000 RCF for 10 min at 4 °C, 75 µL of the supernatant was aspirated and transferred to an autosampler vial (Agilent Technologies, part number 5190–3155), and a volume of 10 μL was injected per run for quantitative analysis by LC-MS/MS.

### 3.5. Analytical Method Validation

The method validation was performed in accordance with the FDA guidance for bioanalytical method validation [33], with modifications as described below.

#### 3.5.1. Selectivity and Linearity

In order to confirm that exemestane and the corresponding internal standard were unaffected by any endogenous substance present in the plasma and to make sure the impact of each analyte in blank plasma is less than 20% of the corresponding analyte at LLOQ levels, the specificity and selectivity of the analytes were evaluated by analyzing chromatograms of extracted analytes in six different batches of blank mouse plasma and comparing the results with the corresponding spiked LLOQ samples. Using a 1/x^2^ weighted least-squares linear regression, exemestane was matched to the corresponding internal standard peak area ratio of eight non-zero calibrators to create the calibration curves.

#### 3.5.2. Precision and Accuracy Matrix Effect, Extraction Recovery, and Carryover

Within a single run of five replicates at each QC level (intra-day) and across four subsequent measurement days (inter-day), the percent coefficient of variation (CV) was used to compute precision. The accuracy was expressed as the percent bias (Bias, %) according to the following equation:

Bias (%) = [(Obs. Mean Conc. − Nominal Conc.)/Nominal Conc.)] × 100%

The acceptance bias of accuracy was set at less than 15% of the nominal values for all QC levels, except for the LLOQ, where it was set at less than 20%. Microsoft Excel version 24.5 was used to conduct statistical analysis.

#### 3.5.3. Matrix Effect, Extraction Recovery, and Carryover

Three duplicate QC samples were tested at low, medium, and high concentration levels to evaluate the matrix effect, hemolysis impact, and extraction recovery. These samples were generated by either spiking the analyte within the extraction reagent, extracted mouse plasma, or using blank mouse plasma, following the described extraction process. For evaluation of the impact of hemolysis on these processes, the analyte was spiked directly into hemolyzed plasma samples.

In order to assess carryover, six analytical runs were carried out where exemestane was measured in an LLOQ sample or a zero-calibration standard right after an ULQ sample was injected. Carryover was considered minimal if the analyte in each blank matrix was below the lower limit of quantification (BLQ) and the percentage deviation from two-thirds of LLOQ samples was within 20% of nominal values.

#### 3.5.4. Stability

##### Short-Term Stability

Triplicates of plasma samples at low, mid, and high QC level concentrations were made and quantified immediately following three or six hours of storage at room temperature (25 °C) or 37 °C in order to test bench-top stability. Exemestane concentrations were compared to the initial injection starting value at time zero. Autosampler stability was recorded after analyzing five replicates of samples after 12 h of storage in a thermostatic autosampler operating at 4 °C using freshly prepared calibrators. Re-injection stability was assessed by storing and reanalyzing the processed samples after 24 h of storage in the autosampler (4 °C) using freshly prepared calibrators.

##### Freeze–Thaw Stability and Long-Term Stability

A separate set of samples, similar to those mentioned above, was prepared and subjected to three freeze–thaw cycles every 24 h (from −80 °C to room temperature) and long-term stability for 5 months at −80 °C. Exemestane concentrations were compared to the initial injection starting value at time zero.

### 3.6. Application in In Vivo Pharmacokinetics Studies

#### 3.6.1. Animal Studies

In vivo pharmacokinetic studies were carried out using age-matched female mice. The FVB background wild-type mice and *Cyp3a*-deficient [*Cyp3a^(−/−)^*] mice that lack all eight murine *Cyp3a* genes on the same background strain were purchased from Taconic Biosciences (Cambridge City, IN, USA) and verified by genotyping test. These mice have been extensively validated [34] and the loss of CYP3A activity was previously confirmed in our lab by liver/intestinal microsomes and in vivo PK studies [35]. The study protocol (201500000101-R2) was approved by the University Laboratory Animal Resources (ULAR) Animal Care and Use Committee at The Ohio State University. The mice were housed in a controlled environment with regulated temperature and lighting and were provided with free access to water and a standard chow diet.

For pharmacokinetic experiments, exemestane was prepared for oral administration by dissolving the drug in DMSO (10%). Subsequently, PEG300 (40%), polysorbate-80 (5%), and saline (45%) were added stepwise to create a 2 mg/mL suspension. Exemestane was administrated orally at doses of 20 mg/kg. Pharmacokinetic studies were performed as described previously [36]. Briefly, serial bleeding of blood samples (~30 μL) was withdrawn at predetermined time points (0.25, 0.5, 1, 2, 4, and 6 h) after drug administration from each mouse (N = 5 per genotype per study; two independent pharmacokinetic studies were performed). The initial three samples were obtained from a submandibular vein using a 5 mm Goldenrod sterile animal lancet. For samples at 2 and 4 h, mice were sedated with 2% isoflurane, and blood was drawn from the retro-orbital venous plexus using heparinized capillary tubes. The 6 h sample was obtained via cardiac puncture using a syringe and needle. All whole blood samples were collected in heparinized tubes, centrifuged at 6000× *g* RCF for 5 min, and the plasma-containing supernatant was collected, snap-frozen on dry ice, and stored at −80 °C until further analysis.

#### 3.6.2. Pharmacokinetic Data Analysis

Plasma concentration–time data were analyzed by non-compartmental methods using Phoenix WinNonlin version 8.0 (Certara, Princeton, NJ, USA) to determine pharmacokinetic parameters in each individual animal. Group averages of the peak concentration in plasma (C_max_) and the area under the plasma concentration–time curve (AUC) extrapolated to infinity were considered as the primary indicators of systemic drug exposure. A two-tailed unpaired t-test was performed between different genotypes to assess the impact of CYP3A deficiency on the C_max_ and AUC of exemestane, and *p*-values of less than 0.05 were used as a cutoff for statistical significance.

## 4. Conclusions

This study reports the development and validation of a sensitive LC-MS/MS method for the quantification of exemestane plasma samples of mice. The method demonstrated acceptable linearity, accuracy, and precision within a concentration range of 0.4–75 ng/mL. Our method was successfully applied to measure concentrations of exemestane in plasma samples collected from mice with varying genetic backgrounds and showed similar exemestane plasma exposure between wild-type mice and mice lacking Cyp3a enzymes. Our results are consistent with the data contained within the exemestane prescribing information, thus allowing DDI predictions to be made with greater confidence. Ongoing research will utilize this method to assess the impact of other metabolizing enzymes and/or transporters involved in the pharmacokinetic profile of exemestane on the connection of these pathways with the development of musculoskeletal side effects caused by this clinically important drug.

## Figures and Tables

**Table 1:** LC-MS/MS method experimental conditions.

List of LC Retention Time and MRM Transitions of Exmastane and [^13^C, D_3_]-Exemestane					
Analyte	Retention Time (min)	Mass Transition (*m/z*)	Collision Energy (V)		Min Dwell Time (ms)
Exemestane	2.82	297.0 → 121.0	20.6		198
		297.0 → 149.0	15.8		198
[^13^C, D_3_]-exemestane	2.81	300.1 → 121.0	19.5		198
		300.1 → 258.9	5.43		198
**LC parameters**					
Mobile phase A	0.1% acetic acid water				
Mobile Phase B	0.1% Formic acid acetonitrile				
Gradient elution program	**Time (min)**		**A%**	**B%**	**Elution change**
	0		70	30	5
	0.5		70	30	5
	3.0		5	95	5
	4.0		5	95	5
	4.1		70	30	5
	4.5		70	30	5
Column temperature	40 °C				
Autosampler temperature	4 °C				
Injection volume Run time	10 μL 4.5 min				
Flow rate	0.3 mL/min				
**MS parameters**					
Sheath gas	25 Arb				
Auxiliary gas	5 Arb				
Sweep gas	1 Arb				
Ion transfer tube temperature	340 °C				
vaporizer temperature	360 °C				
The collision gas argon	1.5 mTorr				
Positive ion spray voltage	3600 V				
Q1 resolution number	0.7 FWHM				
Q3 resolution number	1.2 FWHM				

**Table 2:** Precision and accuracy of the LC-MS/MS method for the determination of exemestane in mouse plasma.

	N	Conc. (ng/mL)	Intra-Assay (CV%)	Inter-Assay (CV%)	Accuracy (Bias%)
LLOQ	20	0.400	7.09	4.64	5.10
LQC	20	1.20	2.19	2.37	3.00
MQC	20	40.0	3.40	2.97	−3.10
HQC	20	65.0	3.63	3.06	−7.80
AULQ (after 10X dilution) ^a^	20	65.0	3.67	4.93	−2.50

Abbreviations: LLOQ, lower limit of quantification; LQC, low-quality control; MQC, medium-quality control; HQC, high-quality control; AULQ, above upper limit of quantification. ^a^ AULQ was diluted with blank mouse plasma to a concentration of 65 ng/mL before deproteination.

**Table 3:** Matrix effect, hemolysis effect, and extraction recovery of exemestane in mouse plasma.

Nominal Con. (ng/mL)		Matrix Effect		Hemolysis Effect		Extraction Recovery	
	N	Mean Matrix Effect (%)	CV (%)	Mean % Nominal	CV (%)	Mean Recovery (%)	CV (%)
LQC (1.2)	3	96.9	3.74	92.9	10.3	99.9	8.80
MQC (40)	3	104	1.93	96.4	5.08	88.4	3.79
HQC (65)	3	108	1.84	102	5.17	90.0	4.18

**Table 4:** (**a**) Autosampler and re-injection stability of exemestane in mouse plasma under various conditions. (**b**). Bench-top stability of exemestane in mouse plasma under various conditions.

(a)									
		Auto Sampler Stability ^a^				Re-Injection Stability ^b^			
Nominal Con. (ng/mL)	N	Mean Deviation (%) of t = 0		CV (%)		Mean Deviation (%) of t = 0		CV (%)	
LQC (1.2)	5	−5.4		4.61		−2.18		2.87	
MQC (40)	5	−6.99		3.38		2.11		4.07	
HQC (65)	5	−0.19		3.9		0.35		2.16	
(**b**)									
		**Bench-Top Stability ^a^**							
		**At 25 °C**				**At 37 °C**			
**Nominal Con. (ng/mL)**	**N**	**3 h**		**6 h**		**3 h**		**6h**	
		**Mean (%) of t = 0**	**CV (%)**	**Mean (%) of t = 0**	**CV (%)**	**Mean (%) of** **t = 0**	**CV (%)**	**Mean (%) of t = 0**	**CV (%)**
LQC (1.2)	3	102	5.43	102	7.31	74.1	5.51	ND	-
MQC (40)	3	98.8	2.33	92.5	2.92	65.5	21.2	2.8	38.7
HQC (65)	3	96.8	2.57	92.5	1.23	62.5	23.9	3.76	49.9

Table (**a**): ^a^ Samples analyzed after 12 h of storage in the autosampler (4 °C). ^b^ Processed samples stored and reanalyzed after 24 h of storage in the autosampler (4 °C). Table (**b**): ^a^ Bench-top storage for 3 h and 6 h at 25 °C (room temperature) and 37 °C.

**Table 5:** Stability of exemestane in mouse plasma under freeze–thaw stability and long-term stability.

Nominal Con. (ng/mL)	Freeze–Thaw Stability (Cycles) ^a^						Long-Term Stability ^b^	
	1st Cycle		2nd Cycle		3rd Cycle			
	Mean % of t = 0	CV (%)	Mean % of t = 0	CV (%)	Mean % of t = 0	CV (%)	Mean % of t = 0	CV (%)
LQC (1.2)	98.1	4.19	95.5	3.13	99.8	4.15	94.8	2.11
HQC (65)	101	1.22	99.8	2.11	98.5	2.62	95.5	5.32

^a^ After three freeze–thaw cycles (from −80 °C to room temperature/ 24 h). ^b^ Storage for 5 months at −80°.

**Table 6:** Pharmacokinetic parameters of exemestane in different genotypes.

Genotype	Sex	Dose (mg/kg)	N	T_max_ (h)	C_max_ (ng/mL)	T_1/2_(h)	AUC _0–6 h_ (ng × h/mL)
Wild-type	Female	20.0	10	0.250	85.0 (±12.0)	2.01	126 (±15.0)
*Cyp3a* ^(-/-)^	Female	20.0	10	0.250	80.0 (±17.0)	2.90	113 (±22.0)

Data represent mean and SEM in parenthesis. Abbreviations: C_max_, peak plasma concentration; AUC, area under the plasma concentration–time curve between time 0 and 6 h; N, number of mice per group.

## Data Availability

The data presented in this study are available on request from the corresponding author.

## Abbreviations

The following abbreviations are used in this manuscript:

LLOQ	lower limit of quantification
LQC	low-quality control
MQC	medium-quality control
HQC	high-quality control
AULQ	above upper limit of quantification
Cmax	peak plasma concentration
AUC	area under the plasma concentration–time curve between time zero and 6 h
N	number of mice per group
